# The dynamic intein landscape of eukaryotes

**DOI:** 10.1186/s13100-018-0111-x

**Published:** 2018-01-24

**Authors:** Cathleen M. Green, Olga Novikova, Marlene Belfort

**Affiliations:** 1 Department of Biological Sciences and RNA Institute, University at Albany, 1400 Washington Avenue, Albany, NY 12222 USA; 2 Department of Biomedical Sciences, School of Public Health, University at Albany, 1400 Washington Avenue, Albany, NY 12222 USA

**Keywords:** Intein, Mobile elements, Horizontal transfer, Endosymbiosis, Sequence similarity network

## Abstract

**Background:**

Inteins are mobile, self-splicing sequences that interrupt proteins and occur across all three domains of life. Scrutiny of the intein landscape in prokaryotes led to the hypothesis that some inteins are functionally important. Our focus shifts to eukaryotic inteins to assess their diversity, distribution, and dissemination, with the aim to comprehensively evaluate the eukaryotic intein landscape, understand intein maintenance, and dissect evolutionary relationships.

**Results:**

This bioinformatics study reveals that eukaryotic inteins are scarce, but present in nuclear genomes of fungi, chloroplast genomes of algae, and within some eukaryotic viruses. There is a preponderance of inteins in several fungal pathogens of humans and plants. Inteins are pervasive in certain proteins, including the nuclear RNA splicing factor, Prp8, and the chloroplast DNA helicase, DnaB. We find that eukaryotic inteins frequently localize to unstructured loops of the host protein, often at highly conserved sites. More broadly, a sequence similarity network analysis of all eukaryotic inteins uncovered several routes of intein mobility. Some eukaryotic inteins appear to have been acquired through horizontal transfer with dsDNA viruses, yet other inteins are spread through intragenomic transfer. Remarkably, endosymbiosis can explain patterns of DnaB intein inheritance across several algal phyla, a novel mechanism for intein acquisition and distribution.

**Conclusions:**

Overall, an intriguing picture emerges for how the eukaryotic intein landscape arose, with many evolutionary forces having contributed to its current state. Our collective results provide a framework for exploring inteins as novel regulatory elements and innovative drug targets.

**Electronic supplementary material:**

The online version of this article (10.1186/s13100-018-0111-x) contains supplementary material, which is available to authorized users.

## Background

**Fig. 1:**
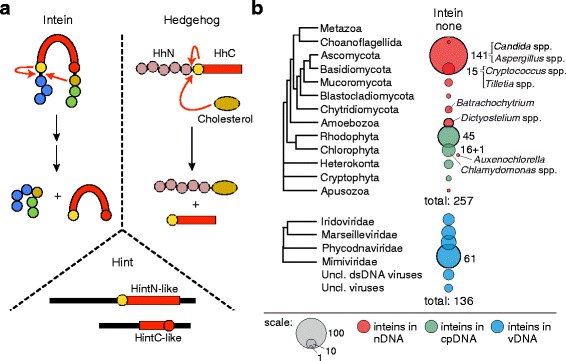
Types of self-splicing protein sequences and their distribution in eukaryotes. **a** Inteins, Hedgehog, and Hint proteins. Inteins are mobile, self-splicing protein elements present across eukarya, bacteria, and archaea. Conserved residues coordinate self-splicing, indicated by red arrows, to ligate the N-extein (blue) and C-extein (green). Hedgehog proteins are found in higher eukaryotes only and are involved in complex developmental processes. They are composed of two domains, HhN and HhC. The HhC domain is analogous to inteins, utilizing a similar mechanism to link cholesterol to HhN (red arrows). *H*edgehog-*int*ein (Hint) domains have cleavage properties similar to either the N-terminus (HintN-like) or C-terminus (HintC-like) of an intein, and are found in both metazoans and lower eukaryotes. **b** A modified phylogenetic tree of eukaryotes was constructed. Scaled circles indicate intein-containing phyla. In fungi, inteins are found in nuclear DNA (nDNA; red circles), in algae in chloroplast DNA (cpDNA; green circles), and in eukaryotic viruses (vDNA; blue circles). Specific intein-containing species are mentioned in the text. Total inteins in each tree are listed

Intervening protein elements, called inteins, are capable of self-excision from precursor polypeptides and ligation of the flanking sequences, termed exteins, to produce the functional host protein [1, 2]. As mobile elements, some inteins also contain a homing endonuclease domain (HEN), allowing movement at the DNA level [3, 4]. Inteins remove themselves from host polypeptides through a process known as protein splicing (Fig. 1a, Intein). This excision occurs in four steps using conserved splice junction residues, often highly reactive nucleophiles, like cysteine, serine, or threonine (Additional file 1: Figure S1).

In addition to inteins, eukaryotes encode other self-splicing proteins that utilize similar chemistry. Hedgehog proteins are structural and functional analogs of inteins, but are involved in cell signaling in higher eukaryotes exclusively [5] (Fig. 1a, Hedgehog; Additional file 1: Figure S2). These essential proteins are synthesized as inactive precursors and undergo nucleophilic attack similar to protein splicing in order to form a functional molecule with cholesterol [6]. Eukaryotes also encode proteins with *H*edgehog-*int*ein (Hint) domains (Fig. 1a, Hint; Additional file 1: Figure S2). Hints employ similar autoproteolytic reactions, either mirroring activity at the N-terminus or the C-terminus of an intein, depending on where peptide cleavage occurs [7]. All three of these eukaryotic self-splicing elements share a canonical Hint fold to promote peptide cleavage reactions [8].

Inteins were originally discovered while studying the vacuolar ATPase (VMA1) of *Saccharomyces cerevisiae* [9, 10]. A central region of the ATPase that had no homology to other known proton pumps was observed. This ‘spacer’ portion, present in the mRNA but not the final protein, turned out to be a 50-kDa self-splicing intein. Since this discovery, thousands of inteins have been documented mainly through sequence-based approaches. A striking outcome has been uncovering inteins across all three domains of life.

In addition to the intein in VMA1, inteins were discovered across other fungal phyla, interrupting diverse genes, such as the pre-mRNA processing factor 8 (Prp8) [11–14]. Experimental data have shown that these fungal inteins are splicing active, at least in foreign exteins, and some were shown to be mobile [15–18]. Additionally, the VMA1 intein was crystallized, providing the first insight into an intein HEN [19]. Chloroplast genes from some algae also contain some of these self-splicing elements [20, 21], and various inteins in eukaryotic viruses were reported [22–25]. The existence of inteins in both prokaryotic and eukaryotic viruses lead to the hypothesis that some inteins may disseminate through this intermediary route [23, 24, 26, 27]. Nevertheless, inteins are found overwhelmingly in the genomes of bacteria and archaea, and a comprehensive picture of the eukaryotic intein landscape is currently lacking [2, 4, 28–31].

A large-scale survey was recently performed to analyze inteins across bacteria and archaea [31]. The results revealed that about half of sequenced archaeal genomes contain at least one intein, whereas a quarter of bacteria are intein-positive among those sequenced. Within this broad distribution, over 60% of proteins containing inteins are involved in replication, recombination, and repair, with 70% localizing to ATP-binding proteins. A similar study investigated where inteins are distributed across bacteria and their phages, providing the first evidence that mycobacteriophages function as facilitators of intein dissemination across mycobacteria [27].

With the recent surge in available sequenced genomes, we next focused on inteins in eukaryotic genes to learn how these inteins might have evolved and spread. Eukaryotic inteins are not only present in nuclear genomes, but in genomes of the chloroplast, and in viral genomes. These inteins frequently interrupt proteins involved in replication, recombination, and repair, as well as RNA processing and energy metabolism, differing somewhat from the tendencies of bacterial and archaeal inteins. Curiously, inteins seem to be enriched in specific lineages of pathogenic fungi, such as *Cryptococcus* spp. More generally, the patterns of eukaryotic inteins suggest important roles in the regulation of the host protein to the potential benefit of the organism in which it resides. Eukaryotic inteins can be classified into three types based on size and presence of a HEN. Apparently, some of these HENs were or are active, based on connections between diverse inteins on a sequence similarity network that is indicative of movement. We discovered that a specific chloroplast intein is a relic of endosymbiosis several billion years ago, while other chloroplast inteins appear to be horizontally transferred by viral vectors. This work suggests a complex, evolving picture of inteins across the eukaryotic mobilome.

## Results

### Eukaryotic inteins are scarce

To assess and characterize the diversity of eukaryotic inteins, we surveyed genomic sequences using BLASTp and previously developed pipelines [31, 32]. At the time of analysis (February 20, 2017), there were 11,001 eukaryotic genomes available through the National Center for Biotechnology Information (NCBI), including 2377 entries for nuclear genomic sequences and 8624 entries for organelle- and plasmid-only sequences. We also analyzed ~ 1500 viral reference proteomes available at ViralZone [33]. The full list of intein-containing genomes and other relevant information is provided in Additional file 2: Table S1 and Additional file 3: Table S2. As an additional point of reference for understanding intein evolution in eukaryotes, we mined genomic sequences for other self-splicing protein elements, including Hedgehog, Hedgehog-like proteins, and *H*edgehog-*i*ntein (Hint) domains (Fig. 1a; Additional file 1: Figure S2 and Table S3). In total, 257 inteins were identified in 231 eukaryotic species, either in their nuclear (nDNA) or chloroplast (cpDNA) genomes, and 136 inteins were found in proteomes from 98 viruses (vDNA) (Fig. 1b).

In general, eukaryotic inteins are scarce compared to bacterial and archaeal inteins. We find that they are widely and sporadically distributed across the eukaryotic tree with the highest number of intein-positive species observed in Fungi, mostly in Ascomycota (141 inteins, or 55% of total; Fig. 1b). Ascomycetes with inteins represent some notable pathogenic Fungi, such as *Candida* spp. [34] and *Aspergillus* spp. [12]. There are 15 inteins (~ 6%) found in Basidiomycota, the close relatives of ascomycetes. Among others, intein-containing basidiomycetes include human pathogens, such as *Cryptococcus neoformans* and *C. gattii* [11, 12], and plant pathogens *Tilletia indica* and *T. walkeri* [35]. A few intein-containing species are identified among other Fungi (Fig. 1b; Table 1 and Additional file 2: Table S1). A noteworthy intein-containing chytrid fungus is *Batrachochytrium dendrobatidis* [12–14], the cause of chytridiomycosis that is devastating amphibian populations [36].

**Table 1:** Most common inteins in nuclear (nDNA), chloroplast (cpDNA), and eukaryotic virus (vDNA) genomes

Protein	Number of inteins	KOG or COG (category)	Description: full name	Distribution
nDNA				
Prp8	104	KOG1795 (A)	pre-mRNA processing factor 8	Pezizomycotina, Agaricomycotina, Ustilaginomycotina, Mucoromycota, Blastocladiomycota, Chytridiomycota, Amoebozoa, Choanoflagellida, Chlorophyta
VMA1	42	KOG1352 (C)	vacuolar ATPase, subunit A	Pezizomycotina, Saccharomycotina, Taphrinomycotina, Puccinomycotina
DdRP	21	KOG0214–0216 (K)	RNA polymerase subunits	Pezizomycotina, Agaricomycotina, Ustilaginomycotina, Bladtocladiomycota, Chytridiomycota, Amoebozoa
ThrRS	5	KOG1637 (J)	Threonyl-tRNA synthetase	Saccharomycotina
GLT	5	KOG0399 (E)	Glutamate synthase	Pezizomycotina, Saccharomycotina
CHS	4	KOG2571 (M)	Chitin synthase	Pezizomycotina
IF2 eIF5B	3	KOG1144 (J)	Eukaryotic translation initiation factor	Chytridiomycota, Glomeromycota
cpDNA				
DnaB	56	COG0305 (L)	DNA replication helicase (DnaB-like)	Rhodophyta, Heterokonta, Cryptophyta
DdRP	12	COG0085 (K)	RNA polymerase subunit beta (RpoB)	Chlorophyta
ClpP	3	COG0740 (OU)	ATP-dependent Clp protease, proteolytic subunit	Chlorophyta
vDNA				
DdDP	67	KOG0969 (L)	DNA polymerases	Phycodnaviridae, Iridoviridae, Mimiviridae, unclassified dsDNA viruses, unclassified viruses
RIR	34	KOG1112 (F)	Ribonucleoside-diphosphate reductases	Phycodnaviridae, Iridoviridae, Mimiviridae, Marseilleviridae, unclassified dsDNA viruses, unclassified viruses
Helicases	20	KOG1123 (KL), KOG2548 (A), KOG0947 (A)	Assorted DNA and RNA helicases	Phycodnaviridae, Marseilleviridae, unclassified dsDNA viruses, unclassified viruses
DdRP	9	KOG0214 (K)	RNA polymerase subunits	Phycodnaviridae, Mimiviridae, unclassified dsDNA viruses
MutS-like	2	KOG0217 (L)	Putative DNA mismatch repair protein	Mimiviridae

A large set of inteins (71 or 28%) was identified in cpDNA from diverse algae and seaweeds (Fig. 1b). Inteins are present in 45 red algae (Rhodophyta), 14 green algae (Chlorophyta), three cryptophytes (Cryptophyta), and nine brown algae and seaweeds (Heterokonta) (Fig. 1b; Table 1; Additional file 2: Tables S1 and Additional file 1: Table S3). Interestingly, no inteins are found in mitochondrial genomes. One green algal species, *Auxenochlorella protothecoides*, has an intein in nDNA. Additionally, inteins are found in the nuclear genomes of some social amoebae (Amoebozoa), as well as in one protozoan species (Apusozoa) (Fig. 1b).

Remarkably, despite intein rarity among eukaryotes, there are species with more than one intein per genome, which might indicate intragenomic spread (discussed below). For example, among Fungi, two inteins are identified in nDNA of an arbuscular mycorrhizal fungus *Rhizophagus irregularis* (Mucoromycota), and a phytopathogenic fungus *Fusarium fujikuroi* (Ascomycota) carries three inteins in its genome.

Among known viruses of eukaryotes, there are 117 inteins across four families: Iridoviridae (14 inteins), Marseilleviridae (20 inteins), Phycodnaviridae (22 inteins), and Mimiviridae (61 inteins). These viral families belong to the nucleocytoplasmic large DNA viruses (NCLDV), also known as order Megavirales, representatives of which are characterized by extremely large genome sizes [37]. An additional 19 inteins are identified in genomes of unclassified viruses (Fig. 1b).

### Intein enrichment in pathogens is genus-specific

**Fig. 2:**
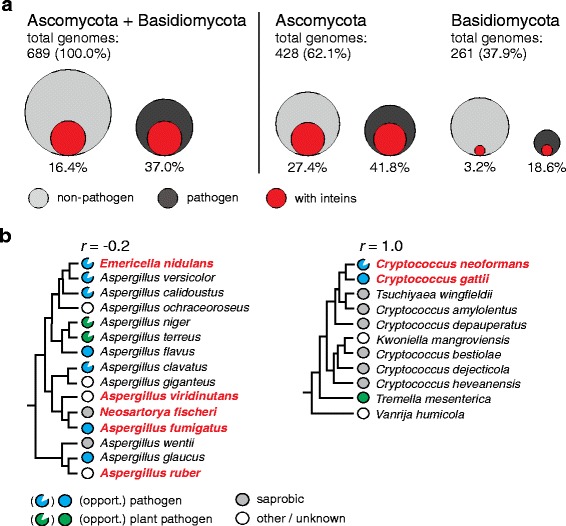
Intein preponderance in pathogenic fungi. **a** Analysis of inteins in pathogens. Two phyla (Ascomycota + Basidiomycota) were analyzed for a propensity of inteins in pathogens (left). Sequenced genomes of non-pathogenic fungi (gray circles) and pathogenic fungi (black circles) were separated and overlaid with the number of intein-positive genomes from each group (red circles). The overall percentage of intein-containing pathogens is 37.0%, higher than the 16.4% of intein-containing non-pathogens. Ascomycota and Basidiomycota were analyzed separately (right), and also show higher number of intein-containing pathogens (41.8% and 18.6% compared to 27.4% and 3.2%, respectively). Out of available sequenced Ascomycota and Basidiomycota, there are more non-pathogenic genomes sequenced than pathogenic, indicating no sequencing bias. Total genomes analyzed are listed. **b** Certain fungal lineages have intein-pathogen correlation. Species within an individual phylum (*Aspergillus*/ascomycete and *Cryptococcocus*/basidiomycete) were analyzed for a correlation of inteins in pathogens. A condensed phylogenetic tree for *Aspergillus* species was constructed and annotated by lifestyle (colored circles). Presence of an intein is indicated by bold and red text*.* While *Aspergillus* contains many inteins, these do not have a preference for pathogenic species, with a negative correlation coefficient (*r* = − 0.2). The phylogenetic tree for *Cryptococcus* shows an absolute correlation (*r* = 1.0), with the only two known pathogens both having inteins

While compiling data on intein distribution, we noticed that inteins are seemingly prevalent in fungal pathogens (Additional file 1: Table S4). To investigate further, we analyzed a distribution of pathogen and non-pathogen representatives among sequenced Ascomycota and Basidiomycota genomes to reveal a potential bias introduced by sequencing pathogenic species (Fig. 2a; Additional file 1: Table S5). It is estimated that roughly 33% of all fungi are pathogens, and a majority of them are ascomycetes, with some basidiomycetes [38]. Out of 689 sequenced genomes from Ascomytoca and Basidiomycota, only 30.2% (208 genomes) are from pathogenic fungi, suggesting that there is no bias in the dataset (Fig. 2a, left, gray versus black circles; Additional file 1: Table S5). There also appears to be no bias towards sequencing genomes from pathogenic species in the separated Ascomycota and Basidiomycota subsets (Fig. 2a, right, gray versus black circles; Additional file 1: Table S5). The overall percentage of intein-containing pathogens from a combined analysis of Ascomycota and Basidiomycota (37.0%) is higher than the percentage of intein-containing non-pathogens (16.4%) (Fig. 2a, left, red circles). The trend remains when looking at the two phyla separately. Ascomycota have 41.8% pathogenic species with inteins versus 27.4% non-pathogenic species with inteins (Fig. 2a, right, red circle). Dramatically, Basidiomycota alone have 6-fold more intein-containing pathogens than non-pathogens, with 18.6% compared to 3.2% (Fig. 2a, right, red circle).

To further investigate this phenomenon, we focused on two intein-containing fungal groups: *Aspergillus*/*Neosartorya* (Ascomycota) and tremellomycetous yeasts (Basidiomycota), including *C. neoformans*, *C. gattii*, and their close relatives (Fig. 2b) [39–42]. We found that within a subset of selected *Aspergillus*, there was no correlation between having an intein and a pathogenic lifestyle, despite several important pathogens having inteins (Fig. 2b, left; Additional file 1: Table S6). On the other hand, within the tremellomycetous yeasts, there was a positive correlation between pathogenic species and intein-containing species. Indeed, *C. neoformans* and *C. gattii* are the only two pathogenic species within the tremellomycetous yeast group, and are the only two intein-containing species in the analysis (Fig. 2b, right; Additional file 1: Table S7). It seems that pathogenic fungi do have a propensity for inteins, but the pattern is variable among specific genera.

### Inteins are found in genes specific to genome type (nDNA, cpDNA or vDNA)

**Fig. 3:**
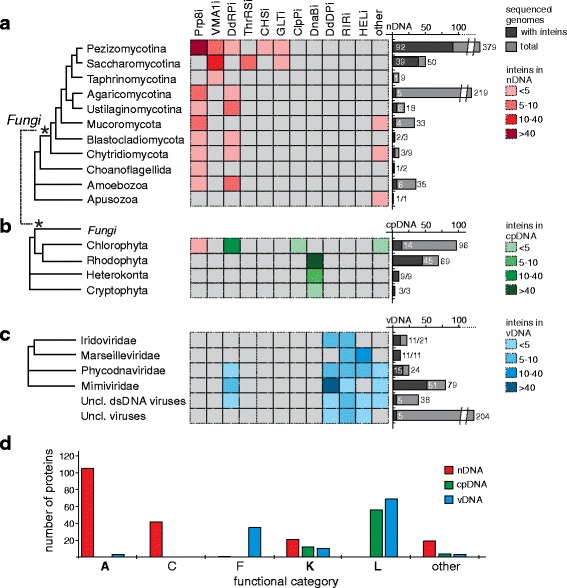
Intein-containing proteins are distinct between nDNA, cpDNA, and vDNA and fall into functional categories. Modified phylogenetic trees of intein-positive species in **a** fungi, choanoflagellates, amoebozoa, and apusozoa, **b** algae and seaweeds, and **c** eukaryotic viruses are presented. The heat maps correspond to the tree and show inteins present in nDNA (red), cpDNA (green), and vDNA (blue). The nDNA inteins are mostly in fungi, overwhelmingly in Prp8, VMA1 and DdRP. One Prp8 intein is found in green algae in nDNA. The cpDNA inteins are in DnaB and ClpP, but are also found in DdRP. The vDNA inteins are present in DdDP, DdRP, HEL, and RIR proteins, but no intein overlap is observed between virus and virus host. Black bars show the number of intein positive genomes relative to the number of sequenced genomes in the phylogenetic category. Extein abbreviations are as follows: Prp8 – pre-mRNA processing factor 8; VMA1 – vacuolar membrane ATPase; DdRP –DNA-directed RNA polymerase; ThrRS – threonyl tRNA synthetase; CHS – chitin synthase; GLT – glutamate synthase; ClpP – ATP-dependent Clp protease, proteolytic subunit; DnaB – DNA helicase; DdDP – DNA-directed DNA polymerase; RIR – ribonucleotide reductase; HEL – helicase. **d** Orthologous group analysis for nDNA, cpDNA, and vDNA classifies intein-containing exteins to functional categories (Additional file 1: Tables S8-S10). nDNA inteins are biased towards category A, RNA processing, from insertions in Prp8. cpDNA and vDNA inteins have bias towards catergory L, or proteins with replication, recombination and repair functions. Functional categories are as follows: A – RNA processing and modification; C – energy production and conversion; F – nucleotide transport and metabolism; K – transcription; L – replication, recombination, and repair

Next, we analyzed the distribution of inteins relative to the protein into which they are inserted (extein) (Fig. 3a–3c; Table 1). Interestingly, there is little or no overlap in exteins between different types of genomes that harbor inteins (Fig. 3a–3c; Table 1). While in nDNA, a majority of inteins are found in Prp8 and VMA1 (Fig. 3a; Additional file 1: Table S8), the replicative helicase DnaB and DNA-dependent RNA polymerase (DdRP) are the most common intein-containing proteins in cpDNA (Fig. 3b; Additional file 1: Table S9). Only red and brown algae contain DnaB inteins, as green algae lack a *dnaB* gene, and the reverse is observed for the ATP-dependent Clp protease, ClpP, and its inteins (Additional file 1: Figure S3). In viral genomes, DNA-dependent DNA polymerases (DdDPs) often harbor inteins (Fig. 3c; Additional file 1: Table S10). The only overlapping intein-containing proteins among all three groups of genomes are DdRPs. However, it should be noted that DdRPs are diverse and genome-specific. Also, the Prp8 intein exists in both nDNA and cpDNA genomes, although its presence in algae might be the result of fungal contamination.

We also sorted exteins into functional caterogies using the KOG (Eu*K*aryotic *O*rthologous *G*roups) database for nDNA and vDNA, or the COG (*C*lusters of *O*rthologous *G*roups) database for cpDNA [43–45] (Fig. 3d; Table 1; Additional file 1: Tables S8-S11). Analysis of functional categories indicates a bias toward category A (RNA processing and modification) for intein-containing proteins from nDNA (108 proteins with intein insertion), and toward category L (replication, recombination, and repair) for exteins from cpDNA (56 proteins) and vDNA (69 proteins) (Fig. 3d; Additional file 1: Table S11). As expected, category K, represented by proteins involved with transcription, is shared by nDNA (21 proteins), cpDNA (12 proteins), and vDNA (10 proteins) for insertions in DdRP exteins. Additionally, category C (energy production and conversion) is prominent among nDNA exteins (42 proteins), and category F (nucleotide metabolism and transport) is overrepresented among vDNA exteins (35 proteins). The other functional categories are less represented (Fig. 3d; Table 1).

### Inteins are located in conserved structure boundaries

**Fig. 4:**
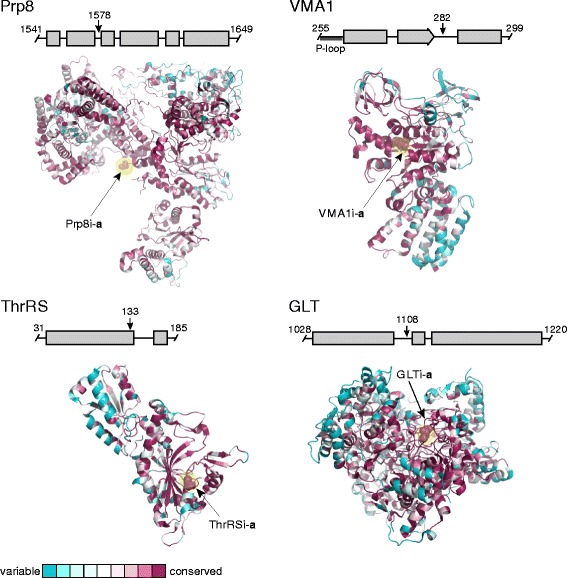
Eukaryotic inteins insert at conserved, structurally flexible regions of host proteins. Intein-containing proteins with PDB structures (Prp8 – 5GMK, VMA1 – 3J9T, ThrRS – 3UGQ, and GLT – 1EA0) were selected to build ConSurf maps, which indicate the degree of conservation after structural alignment. Mauve indicates highly conserved, whereas cyan is more variable as shown in the key. The first residue of the C-extein, shown as spheres, is highlighted in yellow and indicates intein insertion site. Prp8i-**a**, VMA1i-**a**, and GLTi-**a** are in structurally flexible, yet highly conserved sites. The ThrRS intein is inserted in a structured α-helix. Linear cartoons were also generated using Pro-Origami and are shown above the ConSurf maps. Residue numbering indicates the region of protein used in the Pro-Origami model. The black arrow shows intein insertion site and the number corresponds to the highlighted residue in the ConSurf structure. Structure representations are as follows: α-helix – gray rectangle, β-strand – gray arrow, flexible boundary - black line

Inteins are known to occupy sites with specific characteristics that allow splicing besides preceding a nucleophile [46]. Ideally, intein insertion sites must allow proper folding and splicing, and permit the host protein to correctly fold post-splicing [47]. Additionally, inteins are often found in protein active sites and in other conserved, functionally important domains within host proteins [31, 48, 49]. Thus, we examined native insertion sites of the eukaryotic inteins for the properties such as conservation and secondary structure (Fig. 4; Additional file 1: Figure S4). We note that it is a common practice to distinguish inteins based on their extein identity and insertion site [50]. For example, inteins in the Prp8 protein are identified as Prp8i. There are six intein insertion points found in the Prp8 protein, which were designated as **a**-**f** based on the conventional intein insertion site classification scheme [50, 51]. Thus, Prp8i from insertion point **a** is indicated as Prp8i-**a** (Fig. 4).

To determine the local secondary structure of the intein insertion site, we used homology modeling (Additional file 1: Table S13). As demonstrated previously, intein insertion sites are more likely to occur in loop-structure boundary (54% of all sites) than in the middle of a β-sheet or α-helix (31% of sites) [46]. Results of our secondary structure modeling are in agreement with these observations. As demonstrated in Fig. 4 above the 3D structures, the most common eukaryotic inteins Prp8i-**a** and VMA1i-**a**, as well as less widely distributed inteins in threonyl-tRNA synthetase (ThrRSi-**a**) and glutamate synthase (GLTi-**a**), are inserted either in flexible loops, or in close proximity to the loop/α-helix boundary (Fig. 4). Intein insertion in a flexible loop likely allows the intein to fold and splice properly, creating less strain on the host protein.

In order to assess insertion site conservation, the PDB structures of these exteins were uploaded to ConSurf [52], a bioinformatics tool for estimating the evolutionary conservation of amino acid positions in a protein based on the phylogenetic relations between homologous sequences. The ConSurf alignments of nDNA exteins Prp8, VMA1, GLT, and ThrRS reveal they are all in highly conserved sites, as previously noted by several groups [14, 34, 49] (Fig. 4, yellow highlighting; Additional file 1: Figure S4). The structure models also demonstrate the inteins insert at flexible boundaries, often between structured regions. VMA1i-**a** is a clear example, being inserted at a loop between a β-turn and an α-helix.

**Fig. 5:**
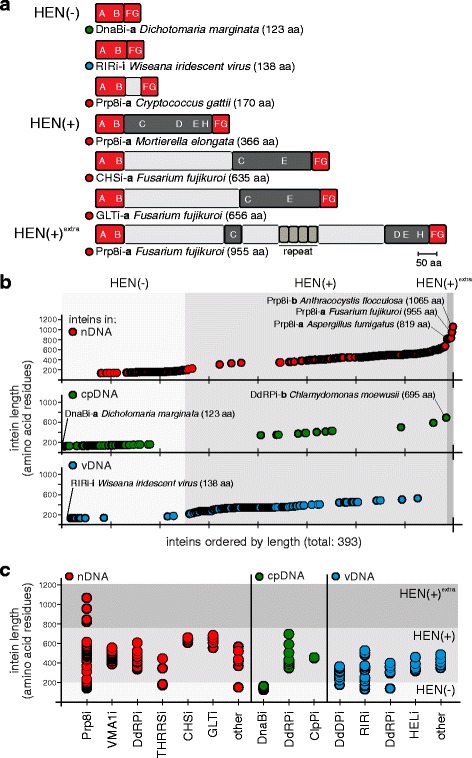
Eukaryotic inteins vary greatly in size. **a** Diversity of eukaryotic intein types. Inteins are classified into three types: HEN(−), HEN(+), or HEN(+)^extra^. HEN(−) inteins contain the four conserved splicing blocks (A, B, F, and G) [50, 79]. Some have linker sequences between block B and F, such as the *C. gattii* Prp8 intein. HEN(+) inteins are full-length, and additionally encode blocks C, D, E, and H for the LAGLIDADG HEN domain. The HEN(+)^extra^ inteins are large, rarely described inteins that have stretches of linker domains or repeat sequences of unknown function. The only examples of HEN(+)^extra^ inteins in eukaryotes are in Prp8*.* **b** All eukaryal inteins in nDNA (red), cpDNA (green), or vDNA (blue) ordered by residue length (totaling 393 inteins). The nDNA inteins show the greatest size diversity, having HEN(−), HEN(+), and the only inteins in the HEN(+)^extra^ category. cpDNA inteins are overwhelmingly HEN(−). vDNA inteins fall between the sizes of nDNA and cpDNA inteins mainly in the range of HEN(+). **c** Inteins in specific exteins cluster by size. When inteins from specific exteins are plotted as a function of residue length, most cluster in the same HEN category, e.g. VMA1i are all HEN(+). Prp8i are the major exception, where inteins range across all three HEN types. Extein abbreviations are listed in Fig. 3 legend

The presence of homing endonucleases (HEN) (see Fig. 5) likely facilitates mobility to conserved regions. Inteins likely home to such positions to decrease the chance of elimination and delay removal, as deletion would require absolute precision to maintain host protein function. This conservation assessment is in line with what has been reported for bacteria and archaea [27, 31, 49]. Two possibilities exist: limitations on mutating the homing site make it difficult for hosts to evolve immunity to the HEN, allowing inteins to propagate as selfish elements, or inteins are functionally important at these conserved sites and are retained to perform yet unknown regulatory roles.

### Eukaryotic inteins vary in size

Many inteins carry a site-specific HEN in addition to the protein splicing domain [4] (Fig. 5a). A HEN renders an intein mobile by introducing a double-strand break into an inteinless allele and initiating a gene conversion event, which results in the copying of the intein coding sequence [4]. This process, known as homing, is thought to be responsible for the horizontal transfer and spread of inteins [53]. Thus, to elucidate the evolutionary dynamics of eukaryotic inteins, it is important to trace the presence/absence of intein-associated HENs and their key features (Fig. 5).

The length of eukaryotic inteins, a simple indicator of the presence of a HEN domain, varies greatly from 123 amino acid residues (DnaBi from red algae *Dichotomaria marginata*) up to 1065 amino acid residues (Prp8i-**b** from grass-infecting smut fungus *Anthracocystis flocculosa*) (Fig. 5a and b). When all identified inteins are plotted by residue length, the resulting plots show a step-wise pattern with varying intein lengths (Fig. 5b; Additional file 1: Figure S5). The lower step, up to ~ 200 amino acid residues, must correspond to inteins that lack HEN domains, referred to as mini-inteins or HEN(−). The middle step between ~200 to 700 amino acids, is likely represented by inteins carrying a HEN or inteins with splicing domains interrupted by a ‘linker’ sequence. These inteins were analyzed for endonuclease motifs and were indeed found to contain LAGLIDADG HENs. The most puzzling aspect of the plot is a group of inteins that are more than 800 amino acid residues in length (Fig. 5b and c). Although there are only a few extra-large inteins and all of them are in Prp8 (Fig. 5c, HEN(+)^extra^), they exist in diverse fungal species, including both ascomycetes (e.g. *F. fujikuroi* and *A. fumigatus*) and basidiomycetes (*An. flocculosa*). In addition to protein splicing domain and HEN, these extra-large inteins carry linker sequences of unknown origin and function, with some of these linkers having repeated motifs (Fig. 5a). Repetitive sequences are common in proteins for structural reasons or may participate in ligand binding [54–56]. The HEN(+)^extra^ inteins were also analyzed for endonuclease type, and many were found to carry LAGLIDADG repetitive motifs (Additional file 2: Table S1 and Additional file 3: Table S2). In general, when inteins in specific exteins are plotted as a function of length, they tend to cluster together in the same HEN category (Fig. 5c).

### Sequence similarity network and phylonetwork of eukaryotic inteins

**Fig. 6:**
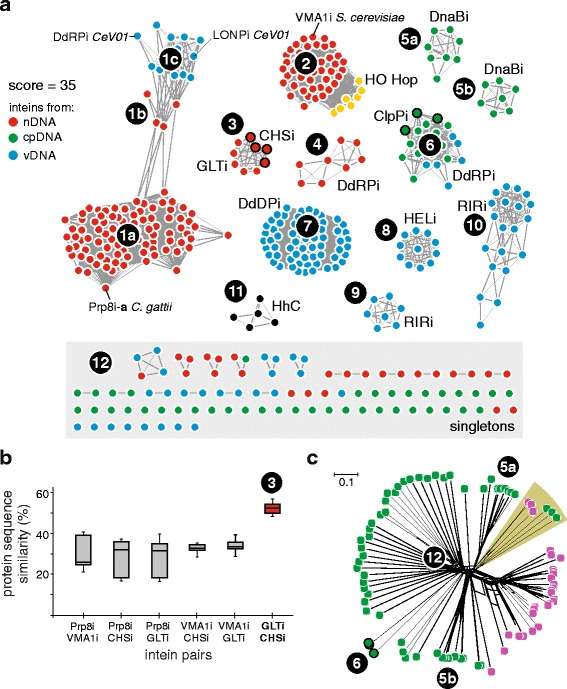
Sequence similarity network reveals inteins cluster by exteins and shows dynamic movement. **a** Eukaryotic intein clustering. The eukaryotic intein network shows relationships between nDNA (red), cpDNA (green), and vDNA (blue) inteins. Network indicates the presence of multiple intein lineages, which mostly correspond to clustering by exteins. Clear examples are Prp8i (1a, 1b) and VMA1i (2), and many viral inteins also cluster by exteins (7, 8, 9, 10). Cases where this pattern is broken represent possible horizontal transfer events (1b, 1c, 3, 6). Some inteins cluster phylogenetically, such as DnaBi from Rhodophyta (5a) or Heterokonta (5b). Hedgehog proteins (black; HhC) do not cluster with any eukaryotic inteins (11), indicating no phylogenetic relationship between Hedgehog and inteins based on sequence, although they are structurally and functionally similar. Hint-containing mating type switching proteins (yellow, HO Hop) cluster with VMA1i (2). Some inteins do not form connections to anything at all (12). **b** Nuclear intragenomic intein transfer. Selected intein pairs were further examined by calculating pairwise similarity percentages and are shown in the box plot. The GLTi and CHSi pair shows an average similarity above 50%, indicative of intragenomic transfer. **c** Endosymbiotic intein transfer. A phylonetwork tree was built in SplitsTree after alignment of cpDNA DnaBi (green) and bacterial DnaBi (pink). A branch of clustering of cpDNA DnaBi and bacterial DnaBi (shaded) suggests that DnaBi in chloroplasts might have been inherited from a cyanobacterial progenitor. Since bacteria also have inteins in ClpP, cpDNA ClpPi were included as a control and they cluster separately (6)

Typically, phylogenetic reconstruction, which focuses on relationships resulting from vertical descent, is used for analysis of homologous genes (or proteins). However, inteins do not follow the rule of strict vertical descent [4, 14, 27, 30, 31], and classic phylogenetic analysis would pose significant limitations to the analysis of intein evolutionary dynamics. Thus, to further elucidate evolutionary dynamics of eukaryal inteins, we built a sequence similarity network (SSN), which does not assume input sequences are homologous (Fig. 6a) [57–60]. The resulting SSN consists of 410 nodes and 5782 edges (links) representing significant relationships between analyzed sequences (Fig. 6a). A majority of the SSN components consists of the sequences originating from the same genomic pool (nDNA, cpDNA or vDNA), and inteins cluster according to their exteins, with a few notable exceptions.

The largest SSN component (Fig. 6a, clusters 1a-1c) is formed by Prp8i (Fig. 6a, cluster 1a and 1b, red) and a group of viral inteins (Fig. 6a, cluster 1c, blue). A smaller cluster of Prp8i (Fig. 6a, cluster 1b), which is separated from the bulk, forms a bridge between a larger cluster of Prp8i (Fig. 6a, cluster 1a) and a cluster of viral inteins (Fig. 6a, cluster 1c). VMA1i (Fig. 6a, cluster 2, red) cluster into a mass of highly interconnected nodes, indicating a high degree of similarity and, together with yeast HO Hop endonuclease (Fig. 6a, cluster 2, yellow) [61], form a large SSN subnetwork. Other SSN components worth mentioning include a large group of viral DdDPi (Fig. 6a, cluster 7, blue), a subnetwork of viral ribonucleotide reductase inteins (RIRi) (Fig. 6a, cluster 10, blue), two smaller SSN components composed of DnaBi from cpDNAs of red algae (Rhodophyta, Bangiophyceae) (Fig. 6a, cluster 5a, green) and brown algae (Stramenopiles, Phaeophyceae) (Fig. 6a, cluster 5b, green). A small group of randomly sampled Hedgehog proteins (C-terminal domain only) (Fig. 6a, cluster 11, black) was included into the analysis and seems to form an outgroup.

There are several SSN components that are formed by inteins located in different proteins. For example, cpDNA inteins, ClpPi and DdRPi from green algae (Chlorophyta) fell within a single connected SSN component (Fig. 6a, cluster 6, green), and also, surprisingly, clustered together with viral DdRPi (Fig. 6a, cluster 6, blue), indicating potential intragenomic intein mobility as well as putative horizontal transfer between cpDNA and vDNA.

One of the most puzzling SSN components consists of fungal glutamate synthase inteins (GLTi) and chitin synthase inteins (CHSi) (Fig. 6a, cluster 3, red). Both GLTi and CHSi are found in genomes of *F. fujikuroi* and *Podospora anserina*. There are also overlaps in distribution among fungal genomes between Prp8i and CHSi, as well as between VMA1i and GLTi. While inteins within Prp8 and CHS are present in *F. fujikuroi* and *Diaporthe helianthi*, the yeast *Debaryomyces hansenii* carries VMA1i and GLTi. Thus, additional inter- and intraspecies pairwise comparative analysis of these inteins was warranted (Fig. 6b; Additional file 1: Table S12). The amino acid sequence similarity between GLTi and CHSi is unusually high, ranging between 48.3% and 56.9%, with an average of 52.3% (Fig. 6b). In contrast, the amino acid sequence similarities in Prp8i-versus-VMA1i, Prp8i-versus-CHSi, Prp8i-versus-GLTi, VMA1i-versus-CHSi, and VMA1i-versus-GLTi comparisons do not exceed 40%, even within the same genome (Fig. 6b). Unusually high similarity between GLTi and CHSi suggests relatively recent intragenomic intein mobility.

Finally, we were intrigued by the large number of DnaBi in cpDNA from taxonomically diverse and evolutionary distant species of eukaryotes (Fig. 1b, Table 1). In bacteria, DnaB is an intein hot-spot [30, 31]. Therefore, we hypothesized that eukaryal DnaBi was likely inherited from the cyanobacterial progenitor of chloroplasts by endosymbiosis [62–64]. Although cpDNA DnaBi show a relatively high degree of protein sequence similarity in pairwise comparisons (data not shown), an overwhelming majority is represented as singletons in the SSN (Fig. 6a, cluster 12, green), which is likely due to difficulties with obtaining meaningful local alignments during the all-against-all BLASTp stage of SSN reconstruction. Thus, to identify possible evolutionary relationships among DnaBi from cpDNA and bacteria, we utilized an alignment-based approach for phylogenetic network inference (Fig. 6c; Additional file 4) [65]. As evident from the resulting phylonetwork (Fig. 6c; Additional file 1: Figure S6), some bacterial and cpDNA DnaBi are distantly related as they cluster together on the same branches, suggesting intein transfer by endosymbiosis.

## Discussion

**Fig. 7:**
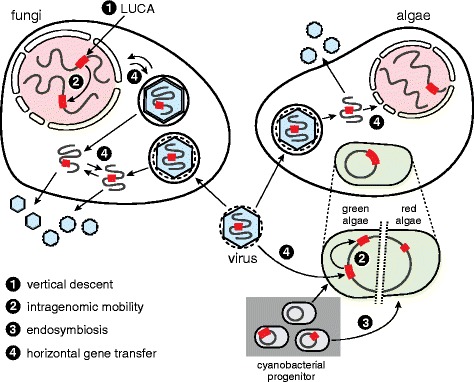
Model for eukaryotic intein distribution and dissemination. Some nuclear inteins present in fungi were likely present in the Last Universal Common Ancestor (LUCA) (1), consistent with intein distribution across all three domains of life. Examples of intragenomic transfer of inteins were also found in both nuclei and chloroplasts (2). The DnaBi within chloroplasts appear to be from reticulate evolution via endosymbiosis (3). Other inteins in fungal cell nuclei and within algae are spread by horizontal gene transfer through eukaryotic viruses that replicate in the cytoplasm (4)

Inteins can execute an autocatalytic protein splicing reaction and perform post-translational modification of a precursor (Fig. 1a). These features make intein-based tools indispensable in modern protein chemistry and bioengineering [66–68]. However, the nature of inteins, their origin, and possible biological roles are still poorly understood, especially in their native context. This work expands our understanding, by providing a comprehensive analysis of intein diversity, distribution, and dissemination in the eukaryotic world. Inteins are found in three genome types: nuclear (nDNA), chloroplast (cpDNA), and viral (vDNA) (Fig. 1b, Fig. 3a-c). Not surprisingly, eukaryotic inteins are inserted in several important, often functionally critical host proteins, matching a global intein trend [14, 27, 31, 49]. The occurrence of inteins in notable human and plant pathogens is a highlight, and will pave the way for further research into intein drug discovery (Fig. 2). Lastly, by applying sequence similarity network (SSN) and phylonetwork analyses, we find evidence for intragenomic mobility, endosymbiotic acquisition, and horizontal transfer of eukaryotic inteins, thus providing an updated picture for how the eukaryotic intein landscape might have evolved (Fig. 7).

### Eukaryotic intein landscape and potential domestication

To gain greater insight into inteins, we previously utilized data mining to investigate the distribution of inteins in prokaryotes, and demonstrated that both bacteria and archaea are relatively intein-rich, with 25% and 50% intein-positive genomes, respectively [31]. In contrast, this study found that fewer than 2% of eukaryotes harbor inteins (Fig. 1b, Fig. 3a-c). The paucity of inteins in eukaryotes suggests that their more complex genomes might have expunged these otherwise invasive elements.

Another possibility for the rarity is that inteins became ‘domesticated’ in eukaryotic genomes and lost some of their features. Indeed, *H*edgehog-*int*ein (Hint) domains are widely distributed among eukaryal phyla (Fig. 1a and Additional file 1: Figure S2) [6, 7]. The function of a majority of these Hint-containing proteins remains unknown, and the essentiality of protein splicing to the host proteins has yet to be established [7]. An evolutionary link of inteins to Hedgehog proteins is also a tantalizing possibility [5, 8]. Although there is only limited sequence similarity between the two self-splicing sequences [5], Hedgehog proteins are structurally analogous to inteins, sharing a characteristic β-strand core known as the Hint module [8]. This common architecture allows both Hedgehog and inteins to undergo post-translational cleavage and ligation by similar trans-esterification reactions to form active molecules. These obvious structural and functional similarities argue in favor of their ancestral relatedness.

### Intein distribution suggests possible functional roles

It was observed that prokaryotic inteins localize to highly conserved sites of vital proteins [14, 31, 34]. In general, this trend holds true for inteins in eukaryotes and their viruses (Fig. 3a-c; Fig. 4). cpDNA and vDNA inteins parallel patterns of extein preference for prokaryotic inteins, with insertions overwhelmingly in proteins involved in replication, recombination, and repair. nDNA inteins are often located in proteins associated with RNA processing, polymerases, and energy production, again critical proteins (Fig. 3d; Table 1). The presence of inteins in essential genes, coupled with their occurrence at different sites within the same protein, argues that inteins may be important to the host protein. On the other hand, these data may also suggest that inteins are successful molecular parasites, by inserting at essential sites where they are less likely to be deleted.

Indeed, recent experimental work has shed light on inteins modulating the host protein through conditional splicing. Inteins in bacteria and archaea have been shown to respond to salt, redox state, temperature, and ssDNA [69–74]. Additionally, evidence from data mining led to the observation that functionally related, but evolutionarily distinct proteins carry inteins, indicating a bias that suggests maintenance due to a regulatory role [31]. There are also often many independent intein insertions into different sites within the same protein or domain. For example, there are 104 varied inteins at different sites in the critical spliceosomal protein Prp8, suggesting several invasion events, and that the inteins are retained because they provide some function. A fascinating hypothesis is that protein splicing acts to regulate RNA splicing, to the selective advantage of the organism.

The finding that inteins are prevalent in human and plant pathogens is also noteworthy (Fig. 2). Given the absence of inteins from metazoan genomes, a pursuit for intein inhibitors as novel antimicrobials is underway [75, 76]. The discovery here of inteins in several more fungal human pathogens strengthens the rationale behind such drug screening efforts. Furthermore, the presence of inteins in agricultural pathogens makes them ripe for exploitation in drug discovery, at a time when fungal diseases are of increasing concern [77].

### Multiple pathways for eukaryotic intein dissemination

To further elucidate eukaryotic intein dynamics, we performed comparative analyses of all known eukaryotic inteins. Phylogenetic analysis of large sets of inteins is often hindered by an inability to produce quality multiple sequence alignments due to a high level of intein sequence diversity. Moreover, there is mounting evidence that horizontal transfer plays a significant role in intein evolution [14, 27, 29–31, 58], but classic phylogenetic analyses focus primarily on vertical descent and traditional phylogenetic trees become inefficient. Here we chose to utilize a SSN-based approach (Fig. 6a), which is less constrained and represents simultaneous interrelationships of all sequences based on their pairwise alignments [46].

Based on the results of our SSN and additional phylonetwork analyses (Fig. 6), we propose a model for intein dissemination in eukaryotes involving vertical inheritance, intragenomic mobility, endosymbiotic and horizontal (extragenomic) acquisitions (Fig. 7). The wide distribution of nDNA inteins and Hint domains among eukaryotes suggests vertical descent from an ancestral sequence as far back as the last universal common ancestor (LUCA) (Fig. 7, pathway 1) [14, 48]. Once in the chromosome, the intein may become fixed in a population and vertically transmit from generation to generation, as is the case for VMA1i (Fig. 6a). Some nDNA inteins may also be mobilized intragenomically (Fig. 7, pathway 2), as is the case for CHSi and GLTi, an event that has been postulated before due to their high sequence similarity (Fig. 6a and b) [48]. Surprisingly, a putative case of intragenomic transfer is also found within the chloroplast genome, suggested by strong relatedness of cpDNA DdRPi and ClpPi (Fig. 6a). To our knowledge, this is the first example of intragenomic intein transfer within an organellar genome.

The largest group of cpDNA inteins, DnaBi, was likely inherited via endosymbiosis from a cyanobacterial progenitor (Fig. 7, pathway 3). Previously, a cyanobacterial DnaBi was shown to have weak sequence similarity with cpDNA DnaBi from a red alga [62]. In the present study, a phylonetwork was reconstructed based on a joint dataset of both cpDNA DnaBi and bacterial DnaBi, and indicates that some of these inteins are distantly related (Fig. 6c). Endosymbiotic acquisition of inteins from bacteria seems reasonable and represents an unprecedented example of transfer of inteins in eukaryotes.

Finally, extragenomic horizontal gene transfer seems to be pervasive and one of the key forces driving eukaryotic intein distribution and diversity (Fig. 7, pathway 4). This is not surprising, given that viruses have been shown to transfer inteins among prokaryotes [23, 24, 27], and research has shown that a family of giant viruses has an ongoing process of exchanging inteins among each other [78]. In algal-like cells (Fig. 7), various instances of intein transmission seem to play a role in the evolution of the current landscape. Here, we again see vDNA inteins clustering with cpDNA inteins (Fig. 6a), suggesting that spread involved viral transfer at some point. Algae prey on bacteria and archaea, and are infected by viruses, providing a place for inteins to transfer directly from bacteria to eukaryotic viruses [25]. The fact that vDNA inteins are found in a class of viruses that replicate in the cytoplasm may allow the opportunity for spread.

## Conclusions

Eukaryotic inteins are scarce compared to their prokaryotic counterparts, although they mimic patterns of distribution that suggest functional importance. These eukaryotic inteins are present in three genome types, nuclear, chloroplast and viral, and these inteins appear to have distinct routes of acquisition. Vertical transmission was primarily observed for fungal nuclear inteins, indicating maintenance since the last common ancestor. HEN-based intragenomic transfer allows these inteins to move to new sites within nuclei, as well as within chloroplasts. Ancient acquisition is also suggested by intein inheritance through endosymbiosis in chloroplast genomes. Finally, horizontal gene transfer spreads inteins across all three genome types, likely mediated by unique eukaryotic viruses that replicate in the cytoplasm. This expansive analysis garnered insight into intein dissemination, and will guide future experiments to investigate intein function and their use as novel drug targets.

## Methods

### Data mining

The first set of full-length precursors and intein protein sequences for eukaryotes was collected from the intein database, InBase [28], and the National Center for Biotechnology Information (NCBI) Protein Database (www.ncbi.nlm.nih.gov/protein/) using previously described pipeline [27, 31]. Next, NCBI BLASTp [32] (blast.ncbi.nlm.nih.gov/Blast.cgi) was used to source additional, mostly unannotated inteins: known intein sequences were used as queries in a series of BLASTp searches against a non-redundant protein database limited to Eukaryota (taxid:2759). The results of the intein search in eukaryotic genomes and other metadata are provided in Additional file 2: Table S1. A similar approach was used to search for viral inteins. However, in addition to mining from NCBI Protein Database, viral inteins were investigated among entries in viral reference proteomes available at ViralZone [33] (https://viralzone.expasy.org/). The results of the intein search in genomes of viruses of eukaryotes are available in Additional file 3: Table S2.

To assess if genomes of pathogenic fungal species were overrepresented in the analysis, fungal species listed on the MycoCosm web portal (http://jgi.doe.gov/data-and-tools/mycocosm/) were examined (February 20, 2017). For each species, a ‘pathogen’ was defined as any species causing plant or human disease. Anything listed as a ‘parasite’, whether of nematodes, plants, or other fungi, were also considered pathogenic for the purpose of this analysis. Any organisms listed as ‘rarely causing disease’ were classified as pathogens. Fungi with unknown medical relevance were not ranked as pathogens.

### Sequence analysis

The primary sequence data obtained for precursor as a whole and intein(s) separately were analyzed further using the following resources. The presence of conserved protein domains and motifs was verified using the Conserved Domain Database (CDD; www.ncbi.nlm.nih.gov/cdd) and Conserved Domain Search Service (CD Search; www.ncbi.nlm.nih.gov/Structure/cdd/wrpsb.cgi) as well as InterPro protein analysis tool (www.ebi.ac.uk/interpro/). KOG annotation for nuclear (nDNA) and viral (vDNA) inteins was performed using protein function annotation by the KOG database tool available at WebMGA server [44, 45] (weizhong-lab.ucsd.edu/metagenomic-analysis/); COG annotation for inteins from chloroplast genomes (cpDNA) was performed using the COG database [43] (www.ncbi.nlm.nih.gov/COG/). The endonuclease size sorting and plotting was done using customized Python scripts, which are available upon request. The CD search tool was utilized to identify which type of endonuclease the HEN(+) and HEN(+)^extra^ inteins encoded. Splicing blocks A, B, F, and G were annotated based on classical classification based on conserved residues [50, 79].

Pairwise and multiple sequence alignments of inteins were performed using Clustal Omega (www.ebi.ac.uk/Tools/msa/clustalo/) unless indicated otherwise. The sequence similarity network (SSN) was generated based on full-length protein sequences of all identified inteins using EFI-EST tool [60] (Additional file 5) (http://efi.igb.illinois.edu/efi-est/) and visualized using Cytoscape [80]. The phylonetwork was built using an alignment of cpDNA DnaBi, ClpPi, and bacterial DnaBi and uploaded into SplitsTree using default settings.

### Secondary structure modeling and insertion site conservation

Intein-containing exteins with known structures were accessed from the Protein Data Bank (www.rcsb.org/pdb/home/home.do). They had the following PDB numbers: Prp8 – 5GMK, VMA1 – 3J9T, ThrRS – 3UGQ, and GLT – 1EA0. The structures have a conserved intein insertion site, despite being from inteinless hosts. Each structure file was truncated to include the domain, or portion of the protein, with intein insertion. All shortened structure PDB files were uploaded to Pro-Origami software (http://munk.csse.unimelb.edu.au/pro-origami/) [81]. For conservation, a single full-length extein sequence was chosen as a query and uploaded to ConSurf [52]. An automatic multiple sequence alignment was performed using MAFFT and homologs were collected from UNIREF90 using a maximum percent identity of 95 and a minimum percent identity of 60. A PDF of the query sequence alone colored according to conservation was downloaded and used for further analysis.

## Additional files

Additional file 1: Supplementary Tables S3-S13 and Supplementary Figures 1–6. This file contains Supplementary Tables S3–13 and Supplementary Figures 1–6 with legends. (PDF 1447 kb)

Additional file 2: Inteins in nDNA and cpDNA. This is a spreadsheet of eukaryotic inteins in nDNA and cpDNA with accession numbers and extein/intein sequences mined in this study. (XLSX 177 kb)

Additional file 3: Inteins in vDNA. This is a spreadsheet of eukaryotic inteins in vDNA with accession numbers and extein/intein sequences mined in this study. (XLSX 80 kb)

Additional file 4: DnaBi alignment. This file contains an alignment of cpDnaBi, bacterial DnaBi, and cpClpPi for Fig. 6c. (FA 57 kb)

Additional file 5: Sequences of all proteins. This file contains all nDNA, cpDNA, and vDNA inteins and HH proteins used in analyses in this study. (TXT 152 kb)

## Abbreviations

CHS: Chitin synthase
ClpP: ATP-dependent Clp protease, proteolytic subunit
COG: Clusters of orthologous genes
cpDNA: Chloroplast DNA
DdDP: DNA-directed DNA polymerase
DdRP: DNA-directed RNA polymerase
DnaB: DNA helicase
DNAtop: DNA topoisomerase
GLT: Glutamate synthase
HEL: Helicases
HEN: Homing endonuclease
HH: Hedgehog
Hint: Hedgehog-intein
IF2 eIF5B: Eukaryotic translation initiation factor 5B
KOG: Eukaryotic orthologous groups
Lon: Lon protease
LUCA: Last universal common ancestor
MutS-like: Mismatch repair protein
NCLDV: Nucleocytoplasmic large DNA viruses
nDNA: Nuclear DNA
PDB: Protein data bank
Prp8: pre-mRNA processing factor 8
RIR: Ribonucleotide reductase
SSN: Sequence similarity network
ThrRS: Threonyl tRNA synthetase
vDNA: Viral DNA
VMA1: Vacuolar membrane ATPase
