# Supplementary material for: The dynamic intein landscape of eukaryotes
Source: Mob DNA. 2018 Jan 24;9:4. doi: 10.1186/s13100-018-0111-x (PMC5784728; doi:10.1186/s13100-018-0111-x)
Supplement: Supplementary file 1 — Supplementary Tables S3-S13 and Supplementary Figures 1–6. This file contains Supplementary Tables S3–13 and Supplementary Figures 1–6 with legends. (PDF 1447 kb) [file 13100_2018_111_MOESM1_ESM.pdf]

ADDITIONAL FILE 3 FOR:

TITLE: The dynamic intein landscape of eukaryotes

AUTHORS: Cathleen M. Green[1], Olga Novikova[1], Marlene Belfort[*,1,2]

AFFILIATIONS:

[1]Department of Biological Sciences and RNA Institute, University at Albany, 1400 Washington Avenue, Albany, NY 12222

[2]Department of Biomedical Sciences, School of Public Health, University at Albany, 1400 Washington Avenue, Albany, NY 12222

[*]Corresponding author:

Marlene Belfort, mbelfort@albany.edu

Co-authors:

Cathleen M. Green, cmgreen@albany.edu

Olga Novikova, onovikova@albany.edu

SUPPLEMENTARY TABLES

Table S3. Distribution of inteins, Hedgehog and Hedgehog-like proteins, and Hint domains among eukaryotes.

Taxon	Subtaxon	Int	HH	Hint
Metazoa		--	522	660
Choanoflagellida		1	--	18
Fungi	Ascomycota	141	--	256
	Basidiomycota	15	--	13
	Mucoromycota	6	--	43
	Blastocladiomycota	2	--	5
	Chytridiomycota	5	--	2
	Zoopagomycota	1	--	--
Amoebozoa		9	--	2
Rhodophyta		45	--	38
Chlorophyta		17	--	30
Streptophyta		--	--	38
Heterokonts		8	--	22
Other Stramenopiles		1	--	22
Alveolates		1	--	51
Cryptophyta		3	--	7
Apusozoa		1	--	3
Other Opisthokonta		4	--	2

Abbreviations: Int – inteins, HH – Hedgehog, and Hint – Hedgehog-like proteins.

Table S4. Inteins in pathogenic fungi: number of sequenced genomes and number of pathogenic fungi among sequenced species.

Taxon	Subtaxon	Total	P
Basidiomycota	Pucciniomycotina	33	15
	Ustilaginomycotina	18	9
	Agaricomycotina	210	19
Ascomycota	Pezizomycetes	16	--
	Orbiliomycetes	2	--
	Eurotiomycetes	106	52
	Dothideomycetes	108	51
	Lecanoromycetes	3	--
	Leotiomycetes	25	4
	Sordariomycetes	106	47
	Xylonomycetes	3	--
	Saccharomycotina	50	8
	Taphrinomycotina	9	3
Glomeromycota		1	--
Mortierellomycotina		4	--
Mucoromycotina		28	9
Zoopagomycotina		2	2
Entomophthoromycotina		3	2
Kickxellomycotina		4	--
Blastocladiomycota		3	1
Chytridiomycota		8	2
Neocallimastigomycota		5	--
Microsporidia		8	8
Cryptomycota		1	1

Abbreviations: Total – total number of sequenced genomes, among which number of pathogenic (P) species was identified.

Table S5. Inteins in pathogenic fungi: distribution of inteins in pathogenic and non-pathogenic Ascomycota and Basidiomycota.

Type	Total	P	NP	Int(+)	P-Int(+)	NP-Int(+)
Ascomycota	428	165 (38.6%)	263 (61.4%)	141 (32.9%)	69 (16.1%)	72 (16.8%)
Basidiomycota	261	43 (16.8%)	218 (83.5%)	15 (5.8%)	8 (3.1%)	7 (2.7%)
Ascomycota + Basidiomycota	689	208 (30.2%)	481 (69.8%)	156 (22.7%)	77 (11.2%)	79 (11.5%)

Abbreviations: Total – total number of sequenced genomes, among which pathogenic (P) and non-pathogenic (NP) species were identified; Int(+) – total number of genomes carrying inteins, P-Int(+) – number of pathogenic species with inteins, NP-Int(+) – number of non-pathogenic species with inteins

Table S6. Inteins in pathogenic fungi: distribution of inteins among pathogenic and non-pathogenic species of closely related *Aspergillus* spp. and *Neosartorya* spp.

Species	Int	P	GS
Aspergillus brevipes	+	--	--
Aspergillus fumigatus	+	+	+
Aspergillus giganteus	+	--	--
Aspergillus ochraceoroseus	+	--	+
Aspergillus rambellii	+	--	+
Aspergillus ruber	+	--	--
Aspergillus viridinutans	+	+	--
Emericella nidulans	+	+ opport.	+
Neosartorya aurata	+	--	--
Neosartorya fennelliae	+	+ opport.	--
Neosartorya fischeri	+	+ opport.	+
Neosartorya fumigata	+	+	+
Neosartorya glabra	+	--	--
Neosartorya quadricincta	+	--	--
Neosartorya spinosa	+	+ opport.	--
Neosartorya udagawae	--	--	+
Aspergillus niger	--	+	+
Aspergillus terreus	--	+	+
Aspergillus flavus	--	+	+
Aspergillus versicolor	--	+ opport.	+
Aspergillus calidoustus	--	+ opport.	+
Aspergillus clavatus	--	+ opport.	+
Aspergillus glaucus	--	--	+
Aspergillus wentii	--	--	+

Abbreviations: Int – species either possess (+) or do not possess (--) inteins, P – indicates pathogenic (+) and non-pathogenic (--) species as well as opportunistic pathogens (+ opport.), GS – genome sequence is available (+) for many species, but some intein-containing species still are not available (--) as full genomic sequence.

Table S7. Inteins in pathogenic fungi: distribution of inteins among pathogenic and non-pathogenic *Cryptococcus* spp. and closely related species.

Species	Int.	P	GS
Cryptococcus neoformans	+	+	+
Cryptococcus gattii	+	+	+
Cryptococcus amylolentus	--	--	+
Cryptococcus depauperatus	--	--	+
Kwoniella mangroviensis	--	--	+
Cryptococcus dejecticola	--	--	+
Cryptococcus heveanensis	--	--	+
Tremella mesenterica	--	--	+
Vanrija humicola	--	--	+
Cryptococcus bestiolae	--	--	+
Tsuchiyaea wingfieldii	--	--	+
Papiliotrema laurentii	+	+ opport.	+

Abbreviations: Int. – species either possess (+) or do not possess (--) inteins, P – indicates if species is pathogenic (+) or non-pathogenic (--) as well as opportunistic pathogens (+ opport.), GS – genome sequence is available (+) for all analyzed species.

Table S8. Inteins from eukaryotic nuclear genomes (nDNA).

Extein	KOG (Category)	Number of Inteins	Description	Distribution
Prp8	KOG1795 (A)	104	RNA processing and modification	Pezizomycotina, Agaricomycotina, Ustilaginomycotina, Mucoromycota, Blastocladiomycota, Chytridiomycota, Amoebozoa, Choanoflagellida, Chlorophyta
VMA1	KOG1352 (C)	42	Energy production and conversion	Pezizomycotina, Saccharomycotina, Taphrinomycotina, Puccinomycotina
DdRP	KOG0214 (K)<br>KOG0215 (K)<br>KOG0216 (K)	21	Transcription	Pezizomycotina, Agaricomycotina, Ustilaginomycotina, Bladtocladiomycota, Chytridiomycota, Amoebozoa
ThrRS	KOG1637 (J)	5	Translation, ribosomal structure and biogenesis	Saccharomycotina
CHS	KOG2571 (M)	4	Cell wall/membrane/envelope biogenesis	Pezizomycotina, Saccharomycotina
GLT	KOG0399 (E)	5	Amino acid transport and metabolism	Pezizomycotina
IF2 eIF5B	KOG1144 (J)	3	Translation, ribosomal structure and biogenesis	Chytridiomycota, Glomeromycota
SRP-alpha	KOG0781 (U)	1	Intracellular trafficking, secretion, and vesicular transport	Glomeromycota
RIR	KOG1112 (F)	1	Nucleotide transport and metabolism	Chytridiomycota
SF3b	KOG0213 (A)	1	RNA processing and modification	Heterokonta
CDC28	KOG0730 (O)	1	Posttranslational modification, protein turnover, chaperones	Apusozoa

Table S9. Inteins from chloroplast genomes (cpDNA).

Extein	COG (Category)	Number of Inteins	Description	Distribution
DnaB	COG0305 (L)	56	Replication, recombination and repair	Rhodophyta, Heterokonta, Cryptophyta
ClpP	COG0740 (OU)	3	Multiple classes	Chlorophyta
DdRP	COG0085 (K)	12	Transcription	Chlorophyta
FtsH-like	COG0464 (O)	1	Posttranslational modification, protein turnover, chaperones	Chlorophyta

Table S10. Inteins from genomes of eukaryotic viruses (vDNA).

Extein	KOG (Category)	Number of Inteins	Description	Distribution
DdDP	KOG0969 (L)	67	Replication, recombination and repair	Phycodnaviridae, Iridoviridae, Mimiviridae, unclassified dsDNA viruses, unclassified viruses
RIR (RNR)	KOG1112 (F)	34	Nucleotide transport and metabolism	Phycodnaviridae, Iridoviridae, Mimiviridae, Marseilleviridae, unclassified dsDNA viruses, unclassified viruses
DdRP	KOG0214 (K)	6	Transcription	Phycodnaviridae, Marseilleviridae, unclassified dsDNA viruses, unclassified viruses
Helicases	KOG1123 (K)<br>KOG2548 (L)<br>KOG0947 (A)	7	Multiple classes; RNA processing and modification	Phycodnaviridae, Mimiviridae, unclassified dsDNA viruses
DNAtop	KOG0355 (B)	1	Chromatin structure and dynamics	Mimiviridae
Gmd	KOG1372 (G)	1	Carbohydrate transport and metabolism	Phycodnaviridae
Lon	KOG2004 (O)	1	Posttranslational modification, protein turnover, chaperones	Phycodnaviridae
MutS-like	KOG0217 (L)	2	Replication, recombination and repair	Mimiviridae
NrdF	KOG1567 (F)	1	Nucleotide transport and metabolism	Unclassified dsDNA viruses

Table S11. Distribution of inteins among functional categories.

Functional Category	Description	Total	nDNA	cpDNA	vDNA
L	Replication, recombination and repair	125	--	56	69
A	RNA processing and modification	108	105	--	3
K	Transcription	43	21	12	10
C	Energy production and conversion	42	42	--	--
F	Nucleotide transport and metabolism	36	1	--	35
J	Translation, ribosomal structure and biogenesis	8	8	--	--
O	Posttranslational modification, protein turnover, chaperones	6	1	4	1
E	Amino acid transport and metabolism	5	5	--	--
M	Cell wall/membrane/envelope biogenesis	4	4	--	--
U	Intracellular trafficking, secretion, and vesicular transport	1	1	--	--
B	Chromatin structure and dynamics	1	--	--	1
G	Carbohydrate transport and metabolism	1	--	--	1

Table S12. Protein sequence similarity (%) between selected inteins.

	1.	2.	3.	4.	5.	6.	7.	8.	9.	10.	11.	12.	13.	14.	15.	16.	17.	18.	19.	20.
1. Prp8i-**a** *Fusarium fujikuroi*																				
2. Prp8i-**a** *Diaporthe helianthi*	13.4																			
3. Prp8i-**a** *Fonsecaea pedrosoi*	15.9	54.0																		
4. Prp8i-**a** *Sporothrix brasiliensis*	35.7	26.2	27.3																	
5. Prp8i-**a** *Sporothrix schenckii*	36.3	25.5	28.2	95.6																
6. VMA1i-**a** *Fonsecaea nubica*	26.2	23.1	25.2	39.0	40.0															
7. VMA1i-**a** *Sporothrix brasiliensis*	26.0	21.2	23.8	39.8	40.6	64.1														
8. VMA1i-**a** *Sporothrix insectorum*	25.9	22.8	25.5	39.6	40.6	69.2	80.6													
9. VMA1i-**a** *Sporothrix schenckii*	25.8	22.2	23.4	40.8	40.2	63.9	95.2	83.2												
10. VMA1i-**a** *Pichia stipitis*	25.4	25.7	25.2	37.9	36.6	61.8	57.9	61.8	59.1											
11. VMA1i-**a** *Debaryomyces hansenii*	22.9	27.9	28.2	36.9	35.8	55.1	49.7	51.1	49.3	62.1										
12. CHSi *Podospora anserina*	31.5	17.3	18.6	36.1	35.7	33.9	32.5	32.7	32.0	32.4	28.8									
13. CHSi *Fusarium fujikuroi*	32.5	16.9	17.3	35.9	35.7	33.7	34.3	33.4	34.3	32.3	31.8	71.5								
14. CHSi *Diaporthe helianthi*	31.2	17.8	18.9	34.4	36.7	35.1	34.4	35.4	35.1	34.6	31.5	65.8	70.9							
15. CHSi *Gaeumannomyces graminis*	32.8	18.4	19.7	36.7	37.2	32.5	30.8	34.4	31.3	32.9	28.5	92.4	70.0	64.6						
16. GLTi *Fusarium fujikuroi*	32.1	16.6	17.8	35.1	36.1	32.9	34.1	33.7	34.3	30.9	28.8	52.7	56.9	49.8	54.2					
17. GLTi *Phaeosphaeria nodorum*	30.4	20.5	17.6	33.7	34.6	34.3	32.3	32.8	31.2	32.9	32.6	51.2	54.8	51.5	50.2	60.7				
18. GLTi *Podospora anserina*	31.9	18.2	18.3	33.1	33.0	32.8	32.8	33.4	32.0	31.2	29.3	51.8	54.8	51.9	53.1	65.0	55.3			
19. GLTi *Debaryomyces hansenii*	31.6	18.3	18.5	39.2	39.0	35.7	36.2	34.3	35.7	35.4	32.6	54.4	55.0	54.0	53.1	61.0	60.8	55.1		
20. GLTi *Pichia guilliermondii*	29.4	18.1	20.6	38.0	39.8	38.0	39.4	39.2	38.0	38.7	35.6	48.8	48.7	49.9	48.3	52.4	53.2	50.7	68.4	
21. DdRPi *Phaeosphaeria nodorum*	25.3	22.8	22.1	34.6	35.6	33.3	34.7	36.6	35.1	34.2	33.8	31.1	31.3	31.1	31.1	30.8	32.1	28.6	33.4	34.0

Table S13. Protein sequences used to build topology models.

Protein (PDB ID)	Domain/Region	Sequence	Length (aa)
Prp8 (5GMK)	Linker	NSKMPTRFPPAVFYTPKELGGLGMISASHILIPASDLSWSKQTDTGIT HFRAGMTHEDEKLIPTIFRYITTWENEFLDSQRVWAEYATKRQEAIQ QNRRLAFEELEGSWDRGIPRISTLFQRDRHTLAYDRGHRIRREFKQYS LERNSPFWWTNSHHDGKLWNLNAYRTDVIQALGGIETILEHTLFKGT GFNSWEGLFWEKASGFEDSMQFKKLTHAQRTGLSQIPNRRFTLWWS PTINRANVYVGFLVQLDLTGIFLHGKIPTLKISLIQIF	274
VMA1 (3J9T)	Catalytic subunit A	GAFGCGKTVISQSLSKYSNSDAIIYVGCGERGNEMAEVLMEFPELYT EMSGTKEPIMKRTTLVANTSNMPVAAREASIYTGITLAEYFRDQGKN VSMIAD	100
ThrRS (3UGQ)	Threonyl-tRNA synthetase, mitochondrial	ATPATMTSMVSQRQDLFMTDPLSPGSMFFLPNGAKIFNKLIEFMKLQ QKFKFGFNEVVTPLIYKKTLWEKSGHWENYADDMFKVETTDEEKEE YGLKPMNCPGHCLIFGKKDRSYNELPLRFSDFSPLHRNEASGALSGL TRLRKFHQDDGHIFC	155
GLT (1EA0)	Glutamate synthase [NADPH], large chain	GTGASPQTSIKFAGLPWEMGLSEVHQVLTLNRLRHRVRLRTDGGLK TGRDIVIAAMLGAEEFGIGTASLIAMGCIMVRQCHSNTCPVGVCVQD DKLRQKFVGTPEKVVNLFTFLAEEVREILAGLGFRSLNEVIGRTDLLH QVSRGAEHLDDLDLNPRLAQVDPGENARYCTLQGRNEVPDTLDARI VADAR	192

SUPPLEMENTARY FIGURE LEGENDS

Figure S1. Expanded protein splicing pathway. Protein splicing occurs in four main steps as discussed in the introduction, and is described here as a canonical class 1 intein. In the first step (1), a conserved residue (usually a Cys or Ser, here shown as Cys in yellow) at the N-terminus of the intein performs a nucleophilic attack on the preceding peptide bond. This results in formation of a thiosester bond between the intein and N-extein. In step two (2), the thioester bond is attacked by the first residue of the C-extein, also a nucleophile (Cys, Ser, or Thr, here shown as Cys in yellow). This results in a branched intermediate species, resolved by a conserved Asn (red) at the C-terminus of the intein in the third step (3). Lastly (4), the N- and C-extein spontaneously reform an ordinary peptide bond, creating the ligated exteins or functional protein. A simplified version of protein splicing is depicted in Fig. 1A.

Figure S2. Eukaryotes encode Hint proteins. A modified phylogenetic tree was constructed to represent taxa with inteins (Fig. 1B). Genomic mining was performed to locate inteins across eukaryotes. For Hint domains, the InterPro database was used to search for Hedgehog/Intein (Hint) domain (IPR028992) in each taxon. Black circles indicate the presence of inteins in each representative phylum; grey circles represent the presence of Hint proteins. Many intein-encoding species also have Hint proteins in their genomes. Some phyla encode more Hint proteins than inteins (e.g. Ascomycota, Alveolates), whereas some have more inteins than Hint (e.g. Basidiomycota, Rhodophyta). Metazoans have both Hint proteins (660) and Hedgehog proteins (black square, 522), and are the only group with Hedgehog proteins.

Figure S3. Extein presence correlates with intein presence. An analysis was done on chloroplast genomes of Chlorophyta, Rhodophyta, Heterokonta, and Cryptophyta to determine if genes for intein-containing exteins exist using a key word search in NCBI Protein Database as described in Novikova et al. 2016 (yellow circle is present and grey circle is absent). Chlorophyta chloroplast genomes do not have a *dnaB* gene, indicating why no inteins were found in DnaB in this group (see condensed heat map on right, Fig. 3B). Chlorophyta do have genes for DdRP and ClpP, and inteins were found in both these

genes (Fig. 3B). Rhodophyta, Hetereokonta, and Cryptophyta encode *dnaB* genes and also have DnaB inteins (Fig. 3B). Curiously, they also encode genes for DdRP, but no inteins were found in these proteins.

Figure S4. Inteins are located at highly conserved sites. Selected intein sequences for Prp8, VMA1, ThrRS, and GLT were submitted to ConSurf servers (Ashkenazy et al. 2016). A multiple sequence alignment was automatically generated and just the protein query is shown with corresponding alignment scores. Purple and cyan indicate high conservation and no conservation, respectively. Each sequence alignment has been trimmed to match the secondary structure diagrams shown in Fig. 4, although the numbers differ because these correspond to the listed sequences, whereas numbers in Fig. 4 correspond to the structure used. Intein insertion is indicated by a black triangle. In general, the inteins are inserted at highly conserved sites in conserved stretches of the host protein.

Figure S5. Inteins sorted by size. Each intein was separated by genome type (nDNA, cpDNA, or vDNA) and then sorted by extein insertion, as in Fig. 5C. The inteins were arranged by length from smallest to largest using a customized Python script, and then plotted to look at the presence or absence of a homing endonuclease domain. nDNA inteins are shown in shades of red. Prp8i show size diversity, while VMAi cluster tightly in the high HEN(+) range. DdRPi are HEN(+) within a broad range. ThrRSi are either HEN(-) or HEN(+). GLTi and IF2i are both HEN(+), but in regions that do not overlap. The same is seen in the plot of SRPαi, RIRi, and CHSi. The last nuclear inteins in SF3bsu1 and CDC28 are HEN(-) and HEN(+), respectively. cpDNA inteins are shown in shades of green. All of the DnaBi are HEN(-) and the remaining inteins in DdRP, ClpP, and FtsH are HEN(+). vDNA inteins sorted by size. DdDPi are both HEN(-) and HEN(+), as are the inteins in DdRP and RIR. Helicase and other inteins group in the HEN(+) category.

Figure S6. Full cpDNA and bacterial DnaBi phylonetwork. A MUSCLE alignment of a subset of bacterial DnaBi and all of the chloroplast DnaBi was performed using UGENE.

The three ClpPi were also included as a control. The alignment was uploaded to SplitsTree and a phylonetwork was constructed under basic parameters. The phylonetwork shows a single branch of clustering of bacterial DnaBi and chloroplast DnaBi, indicating similarity and possible endosymbiotic transfer. The simplified version of the phylonetwork is shown in Fig. 6C. The ClpP inteins do not branch with any other inteins. Most chloroplast DnaBi and bacterial DnaBi do not cluster, suggesting a long evolutionary timescale since the event happened, in line with the timescale of endosymbiosis.

SUPPLEMENTARY FIGURES

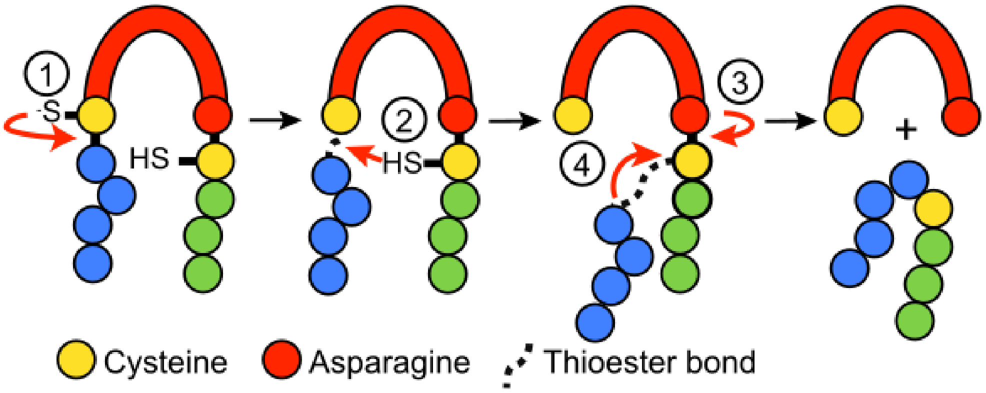

Figure S1. Expanded protein splicing pathway.

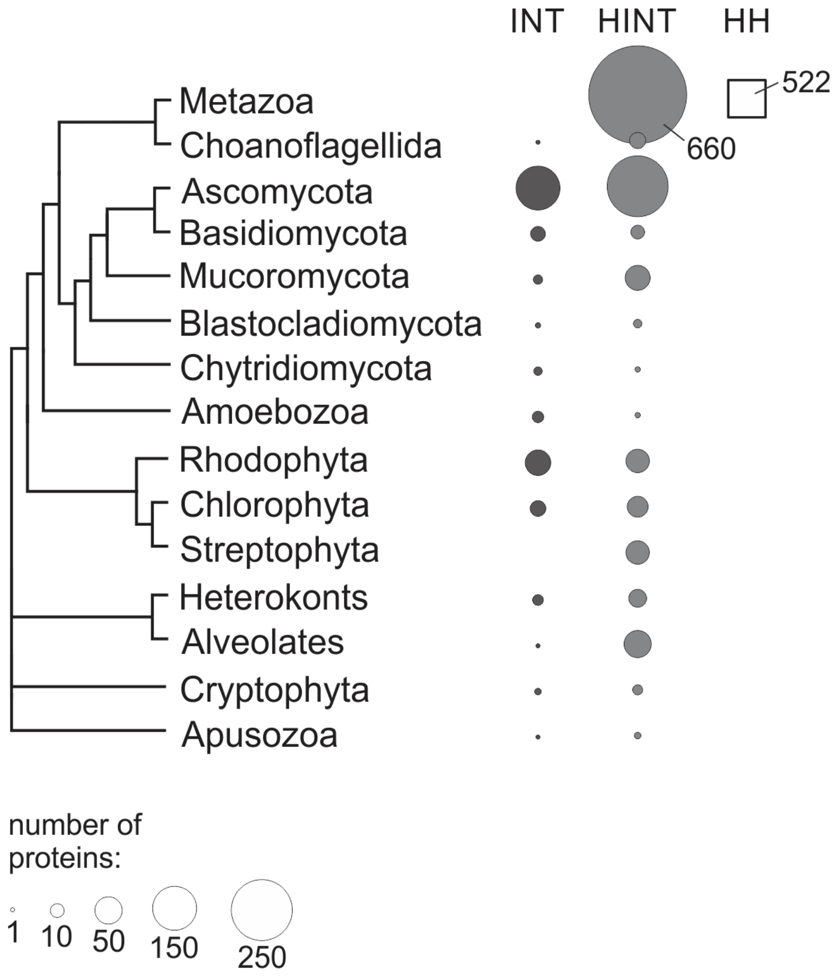

Figure S2. Eukaryotes encode Hint proteins.

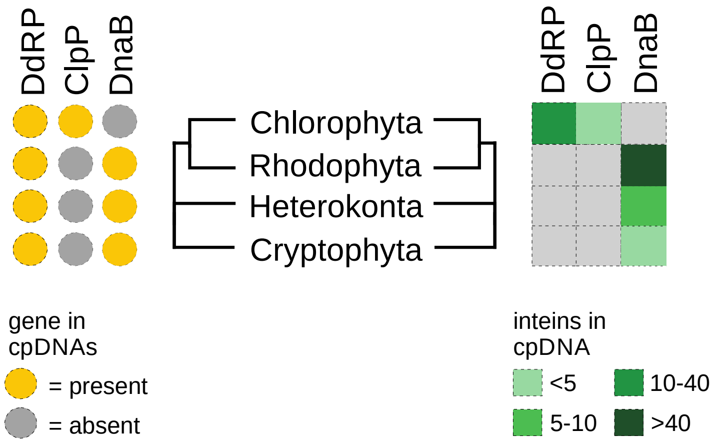

Figure S3. Extein presence correlates with intein presence.

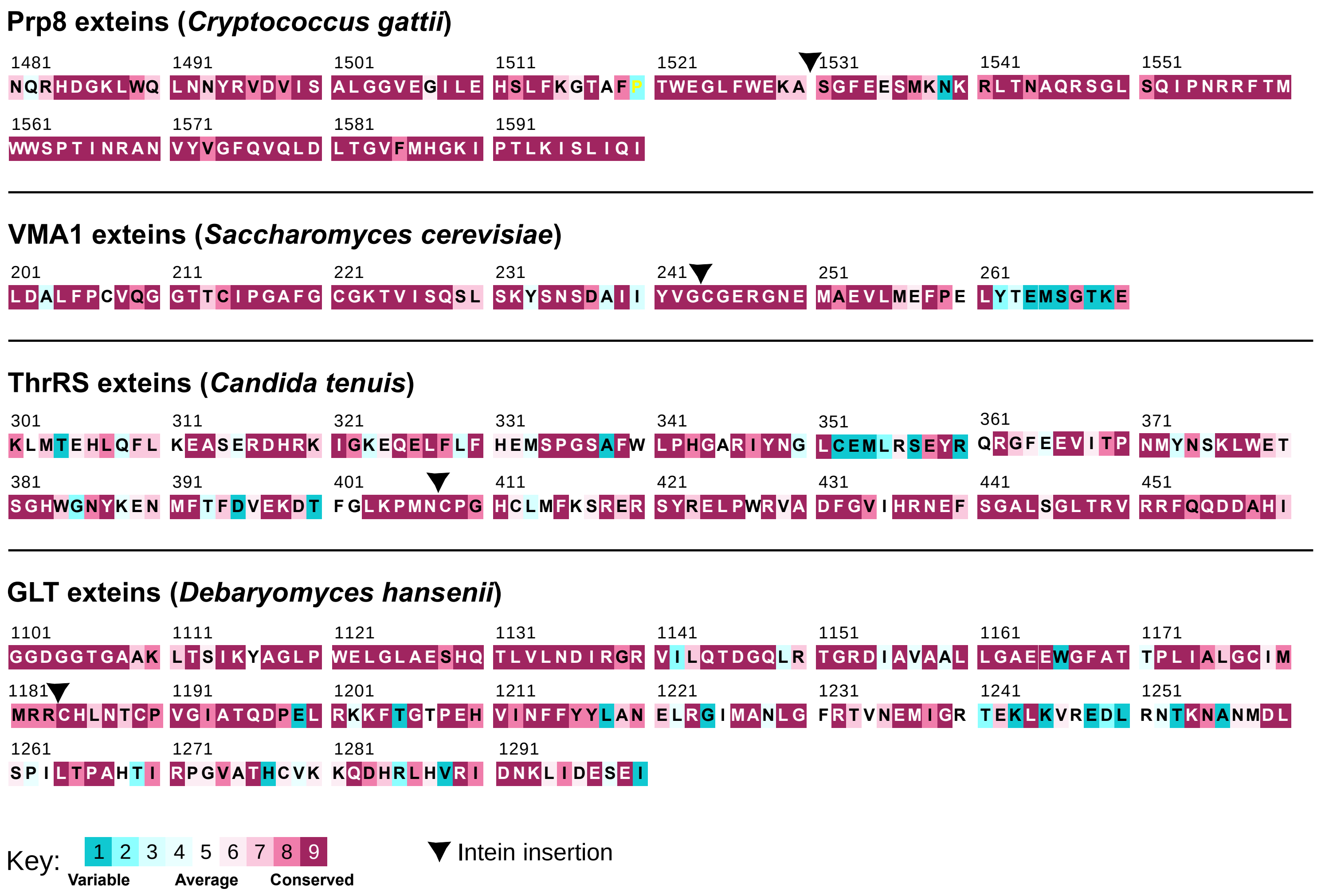

Figure S4. Inteins are located at highly conserved sites.

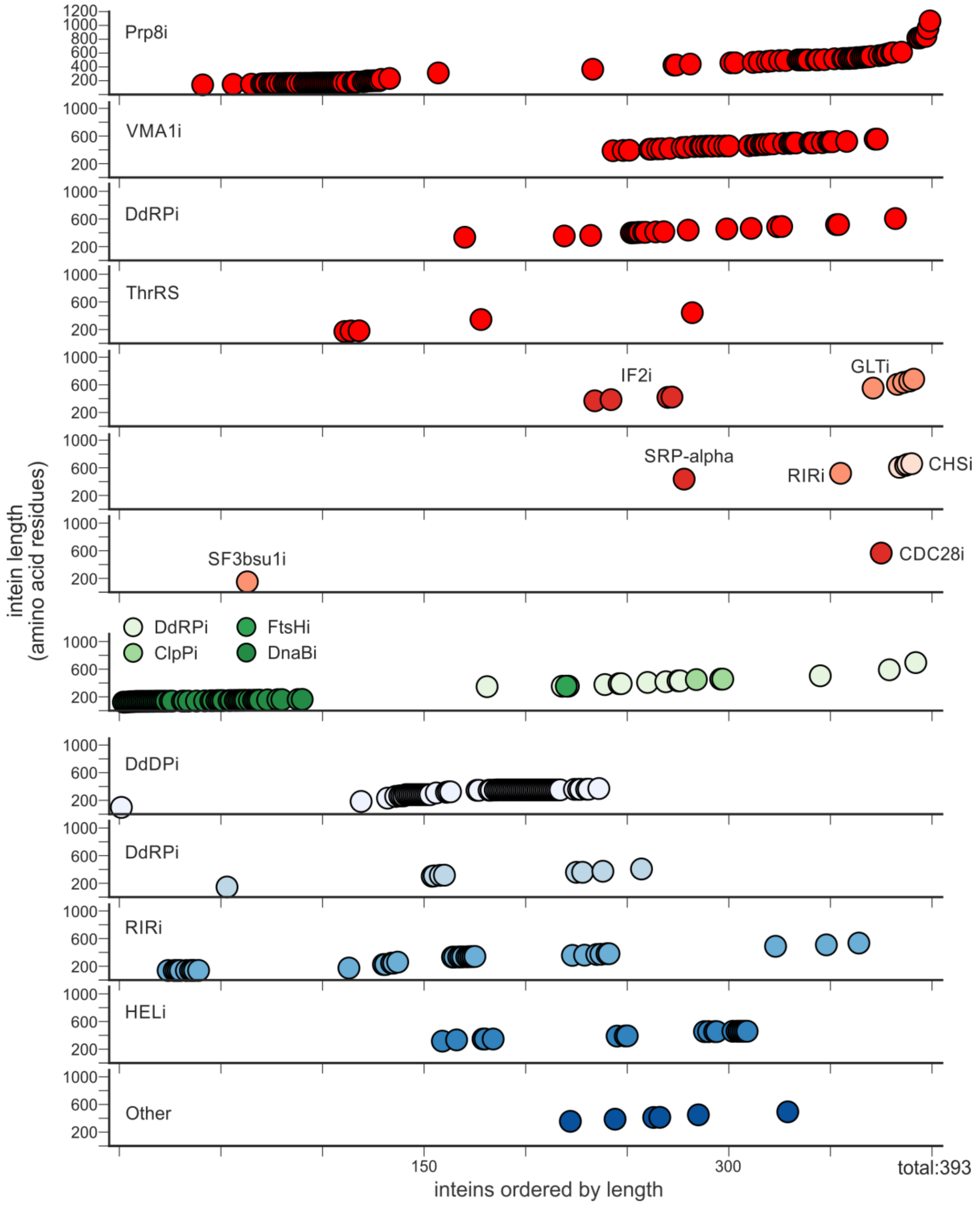

Figure S5. Inteins sorted by size.

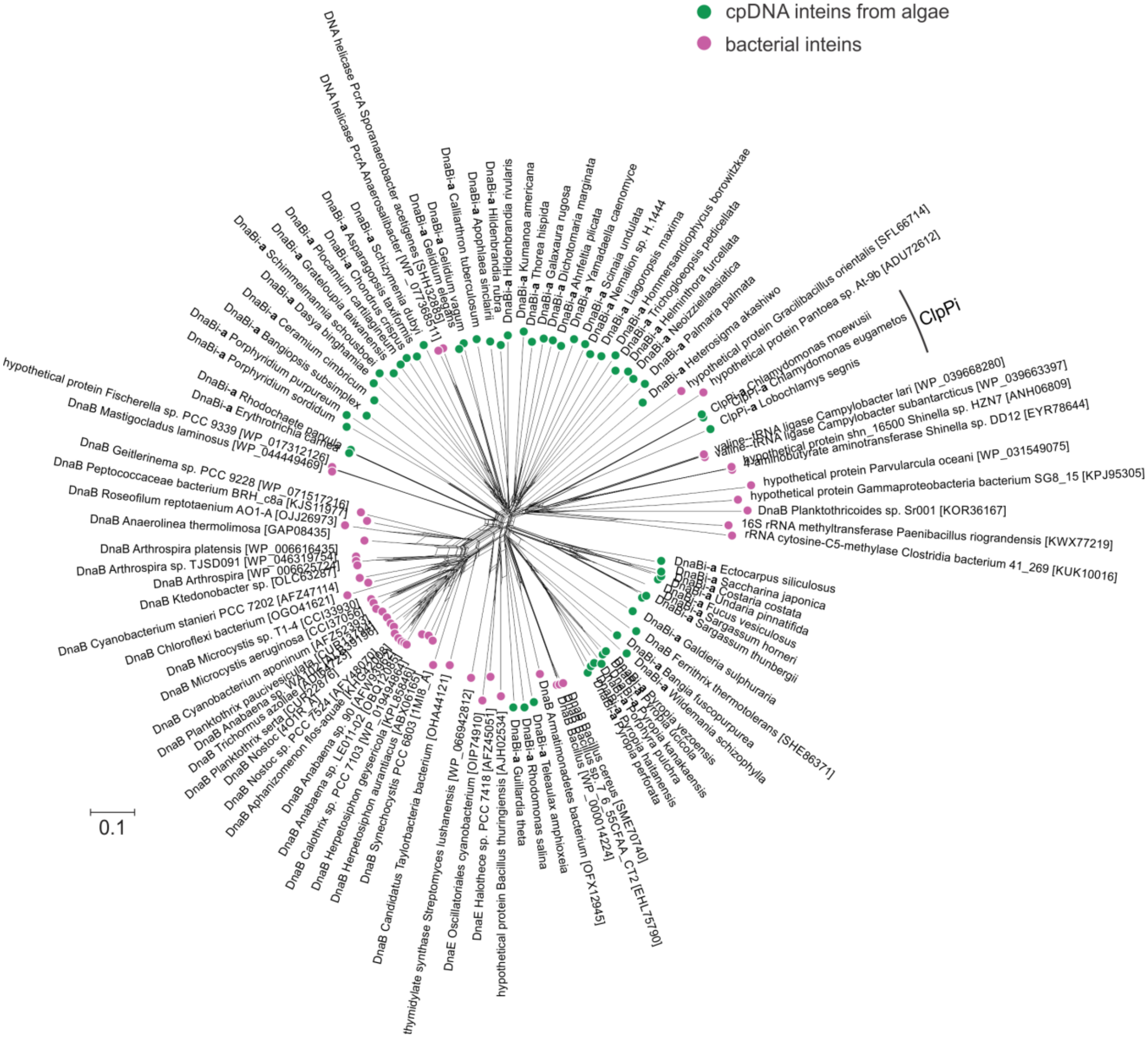

Figure S6. Full cpDNA and bacterial DnaBi phylonetwork.

REFERENCES:

Ashkenazy, H, S Abadi, E Martz, O Chay, I Mayrose, T Pupko, N Ben-Tal. 2016. ConSurf 2016: an improved methodology to estimate and visualize evolutionary conservation in macromolecules. Nucleic Acids Res 44:W344-350.

Novikova, O, P Jayachandran, DS Kelley, Z Morton, S Merwin, NI Topilina, M Belfort. 2016. Intein Clustering Suggests Functional Importance in Different Domains of Life. Mol Biol Evol 33:783-799.
